# Are late hernia mesh complications linked to Staphylococci biofilms?

**DOI:** 10.1007/s10029-022-02583-0

**Published:** 2022-03-14

**Authors:** P. Patiniott, A. Jacombs, L. Kaul, H. Hu, M. Warner, B. Klosterhalfen, A. Karatassas, G. Maddern, K. Richter

**Affiliations:** 1 Surgery Department, The Queen Elizabeth Hospital and Basil Hetzel Institute for Translational Health Research, The University of Adelaide, Adelaide, Australia; 2 Macquarie University Hospital, Macquarie University, Sydney, Australia; 3 Institute of Pharmaceutical Sciences, Department of Pharmaceutical Technology and Biopharmacy, University of Freiburg, Freiburg, Germany; 4 Faculty of Medicine, Health and Human Sciences, Macquarie University, Sydney, Australia; 5 Microbiology and Infectious Diseases Directorate, SA Pathology, Adelaide, Australia; 6 Faculty of Health and Medical Sciences, The University of Adelaide, Adelaide, Australia; 7 Infectious Diseases Unit, Central Adelaide Local Health Network, Adelaide, Australia; 8 MVZ für Histologie, Zytologie und Molekulare Diagnostik Düren GmbH, Düren, Germany; 9 Institute for Photonics and Advanced Sensing, The University of Adelaide, Adelaide, Australia

**Keywords:** Hernia, Biofilms, Implant infection, Mesh failure, Mesh complication, Chronic pain, *Staphylococcus aureus*

## Abstract

**Purpose:**

The purpose of this study was to investigate the link between bacterial biofilms and negative outcomes of hernia repair surgery. As biofilms are known to play a role in mesh-related infections, we investigated the presence of biofilms on hernia meshes, which had to be explanted due to mesh failure without showing signs of bacterial infection.

**Methods:**

In this retrospective observational study, 20 paraffin-embedded tissue sections from explanted groin hernia meshes were analysed. Meshes have been removed due to chronic pain, hernia recurrence or mesh shrinkage. The presence and bacterial composition of biofilms were determined. First, specimens were stained with fluorescence in situ hybridisation (FISH) probes, specific for *Staphylococcus aureus* and coagulase-negative staphylococci, and visualised by confocal laser scanning microscopy. Second, DNA was extracted from tissue and identified by *S. aureus* and *S. epidermidis* specific PCR.

**Results:**

Confocal microscopy showed evidence of bacterial biofilms on meshes in 15/20 (75.0%) samples, of which 3 were positive for *S. aureus*, 3 for coagulase-negative staphylococci and 9 for both species. PCR analysis identified biofilms in 17/20 (85.0%) samples, of which 4 were positive for *S. aureus*, 4 for *S. epidermidis* and 9 for both species. Combined results from FISH/microscopy and PCR identified staphylococci biofilms in 19/20 (95.0%) mesh samples. Only 1 (5.0%) mesh sample was negative for bacterial biofilm by both techniques.

**Conclusion:**

Results suggest that staphylococci biofilms may be associated with hernia repair failure. A silent, undetected biofilm infection could contribute to mesh complications, chronic pain and exacerbation of disease.

## Introduction

The presence of bacterial biofilms in implanted hernia meshes and their impact on hernia repair outcomes has not been investigated to date. Bacterial biofilms are associated with chronic infections, exacerbation of disease, failure of standard treatments, prosthetic device failure and other major complications. Therefore, we hypothesised that biofilms significantly affect hernia repair surgery outcomes, even if an infection is absent. The effects of biofilms resulting in a reduction of mesh effective porosity and subsequent limitation of mesh–tissue integration may present a plausible explanation for delayed “mesh infection”, chronic seroma, chronic pain, and failure of hernia repair [1].

Biofilm has been defined as an “aggregate of microorganisms in which cells that are frequently embedded within a self-produced matrix of extracellular polymeric substance (EPS) adhere to each other and/or to a surface” (International Union of Pure and Applied Chemistry) [2]. Bacterial biofilms are formed when bacteria convert from their planktonic (“free-floating”) genetic phenotype to a sessile genetic phenotype [3]. This enables the bacteria to form communities composed of single or multiple species of microorganisms attached to surfaces that interact with each other and their environment conferring a significant survival advantage [4].

Once the biofilm producing phenotype has been activated the bacteria begin to produce a protein–polysaccharide-rich matrix of EPS which encloses them [3, 5]. The biofilm matrix offers the resident bacteria a three-dimensional structure that provides mechanical stability and physical protection from external stressors. The EPS matrix can prevent or limit the activity of immune cells and therapeutic compounds entering or diffusing through the matrix, hence providing a protected haven for bacteria to survive and reproduce [5]. Conversely, the EPS matrix facilitates bacterial communication, important for reproduction and survival, and promotes exchange of genetic information, including antibiotic-resistance genes [5, 6]. Hence antibiotic-resistance genes often rapidly spread within biofilms, making bacteria up to 1000-fold more resistant to antibiotics compared to planktonic cells [6, 7]. The physical barrier and swift integration of antibiotic-resistance genes make the eradication of biofilms challenging even with best medical interventions [3, 8, 9].

Device-related infections were the first clinical infections to be identified as having a biofilm aetiology and show that biofilm formation can be facilitated by the host inflammatory response because host inflammatory molecules facilitate adhesion to the surface of the device [10]. Biofilm formation on medical implants has even led to the characterisation of a new infectious disease called chronic polymer-associated infection [11, 12].

Skin flora, including *Staphylococcus aureus* and skin commensal coagulase-negative staphylococcal species, in particular *Staphylococcus epidermidis*, are frequently linked to biofilm formation on orthopaedic joint prostheses and breast implants [13–15]. Implant contamination has been shown to occur mainly at time of insertion, when the risk of implant contact with patient’s own skin is highest [13, 14]. It is therefore reasonable to expect the above commensal organisms to also be present in hernia mesh-related biofilm; accordingly, this consideration was factored into our study design.

In this study, our main objective was to investigate whether there was evidence of bacterial biofilm on mesh used in inguinal hernia repairs, that had been explanted due to complications including recurrence and chronic pain. Moreover, we aimed to identify the bacterial species on mesh with a focus on *S. aureus* and coagulase-negative staphylococci.

## Materials and methods

All chemicals have been purchased from Sigma-Aldrich, unless stated otherwise.

### Sample preparation and microscopic analysis

Twenty deidentified paraffin-embedded tissue sections from explanted groin hernia mesh in patients with chronic pain were provided by Professor Bernd Klosterhalfen, MVZ für Histologie, Zytologie und Molekulare Diagnostik Düren GmbH, Düren, Germany.

Each specimen was sectioned into 5 µm slices using a Microm HM 325 microtome (GMI, USA) and duplicate sections were placed on adhesion slides for microscopy (Menzel Gläser SuperFrost® Plus, Thermo Fisher Scientific, USA). The specimens were heated to 60 °C in a heat bath, deparaffinised in xylene and rehydrated in serial ethanol and demineralised water before heat fixation. Each mesh specimen was stained with peptide nucleic acid fluorescence in situ hybridisation (PNA-FISH) probes from AdvanDx (OpGen®, Denmark) specific for *S. aureus* and coagulase-negative staphylococci rRNA using the manufacturer’s protocol.

The slides were mounted with ProLong™ Gold antifade mounting medium containing the DNA stain DAPI (Invitrogen, Thermo Fischer, USA) and imaged with an Olympus FV3000 laser scanning confocal microscope (Adelaide Microscopy, Australia) for analysis and detection of bacterial biofilm in the explanted hernia mesh tissue. The resulting images were independently reviewed by two researchers and a consultant clinical microbiologist. The findings were subsequently correlated with the clinical and mesh information.

### DNA extraction from formalin-fixed paraffin-embedded (FFPE) tissue and real-time PCR

A thin slice of mesh FFPE tissue was cut with a sterile razor blade and deparaffinated using xylene and ethanol. Paraffin-cleaned tissue was digested in 1 mg/ml proteinase K at 56 °C for 2 h or overnight (until the sample has been completely lysed), followed by incubation with 0.5 mg/ml lysozyme at 56 °C for 1 h and 0.5 mg/ml proteinase K at 56 °C for an additional hour. The cell lysate was incubated at 90 °C for 1 h to break DNA cross-links. DNA was extracted using a High Pure FFPET DNA isolation kit (Roche, product 06650767001) according to the manufacturer’s instructions.

Real-time PCR targeting 16S rRNA genes was performed to detect bacterial species. *S. aureus* was identified using *S. aureus* species-specific primers 5′-GCGATTGATGGTGATACGGTT-3′ and 5′-AGCCAAGCCTTGACGAACTAAAGC-3′ targeting the *nuc* gene [16]. *S. epidermidis* was detected using *S. epidermidis* species-specific primers 5′-GGAAGTTCTGATAATACTGCTG-3′ and 5′-GATGCTTGTTTGATTCCCTC-3′ targeting the *icaA* gene [17].

Twenty microlitre PCR mixture containing 1X PowerUp SYBR Master Mix (Applied Biosystems, Cat# A25741), 400 nM forward and reverse primers and 100 ng DNA template was processed under the following conditions in the ViiA™ 7 Real-time PCR System (Applied Biosystems): an initial temperature of 50 °C for 2 min followed by 95 °C for 10 min, then 40 cycles of 95 °C for 15 s and 60 °C for 1 min. *S. aureus* or *S. epidermidis* DNA was used as positive control, and nuclease-free water was used as no template control in the PCR.

### Ethics statement

This study was reviewed by the Central Adelaide Local Health Network Human Research Ethics Committee, Australia and was deemed to meet the requirements of the National Statement on Ethical Conduct in Human Research 2007 (updated 2018). The study was exempt from Ethics approval as it carries only negligible risk and involves the use of existing data that contain only non-identifiable data about human beings.

## Results

This was a retrospective observational study of 20 explanted mesh tissue specimens that were analysed for evidence of *S. aureus* and coagulase-negative staphylococci/*S. epidermidis* biofilm infection with microscopy and DNA identification. Each specimen was an explanted polypropylene-based mesh from various manufacturers, which had previously been inserted as part of an open or laparoscopic inguinal hernia repair or incisional hernia repair.

The majority of patients were male (*n* = 18; 95.0%) with a median age of 50 years and 6 months. Most of the hernia repairs were done with an open-Lichtenstein technique (*n* = 13; 65.0%), laparoscopic (TEPP or TAPP) hernia repair (*n* = 4; 20.0%), Plug mesh repairs (*n* = 2; 10.0%) and open incisional hernia sublay repair (*n* = 1; 5.0%). The median time from hernia repair to explantation was 24.5 months (range 8–72 months) and there were a range of polypropylene mesh products explanted as detailed in Table 1. All patients had one or more indications for explantation including chronic pain (*n* = 14; 70.0%), hernia recurrence (*n* = 9; 45.0%) and mesh shrinkage (*n* = 4; 20.0%). Two patients (10.0%) had both hernia recurrence and mesh shrinkage or both hernia recurrence and chronic pain. One patient (5.0%) had both chronic pain and mesh shrinkage and one patient (5.0%) had hernia recurrence, chronic pain and mesh shrinkage. No patient had documented infective symptoms.

**Table 1:** Excised mesh products and associated biofilm identification by confocal microscopy and PCR

Product	Number of probes (%)	Procedure type (number)	Mesh porosity	Biofilm/bacterial identification		
				Imaging and PCR positive	Imaging or PCR positive	Imaging and PCR negative
Ultrapro	7 (35.0%)	Open (5), laparoscopic (1), incisional (1)	2.0–4.0 mm	3	3	1
Prolene/Soft Prolene	4 (20.0%)	Open (4)	0.8 mm	4		
Optilene	2 (10.0%)	Open (2)	1.0–3.6 mm	1	1	
PerFix Plug	2 (10.0%)	Plug (2)		2		
Vypro II	2 (10.0%)	Open (1), laparoscopic (1)	1.0–2.5 mm	2		
Adhesix	1 (5.0%)	Laparoscopic (1)	Macroporous		1	
Atrium	1 (5.0%)	Laparoscopic (1)	0.8 mm		1	
TiMesh	1 (5.0%)	Open (1)	> 1.0 mm	1		
				Total bacterial presence		
				13 (65.0%)	6 (30.0%)	1 (5.0%)

### Bacterial biofilm identification

Each explanted mesh was assessed for the presence of bacteria with two techniques: (i) PNA-FISH combined with confocal microscopy using probes specific for *S. aureus* and coagulase-negative staphylococci for the detection of staphylococci biofilms, and (ii) PCR with species-specific primers targeting *S. aureus* and *S. epidermidis*, which are the most common bacteria associated with implant biofilm infections (Table 1).

### Confocal microscopy

**Fig. 1:**
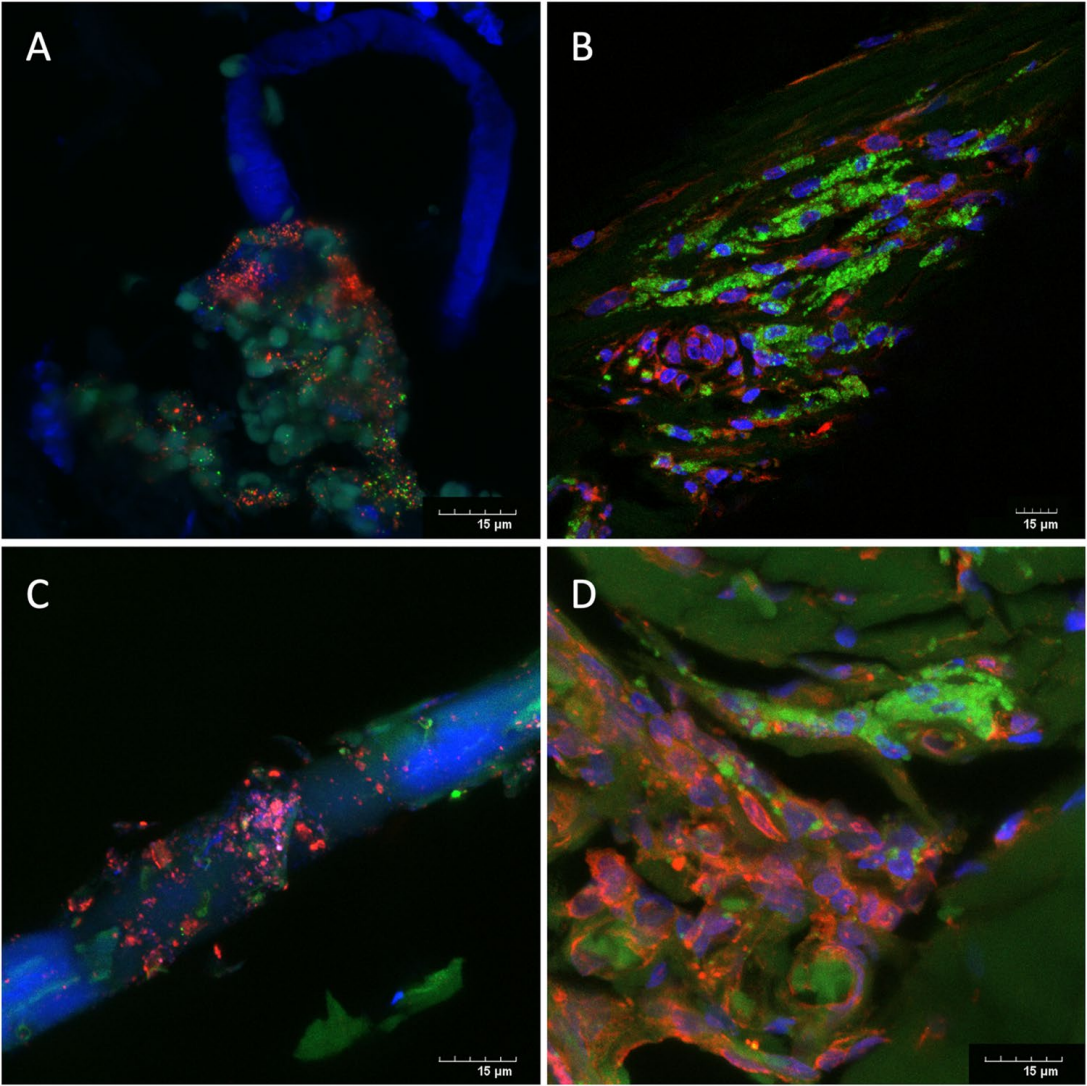
Confocal microscopy images of PNA-FISH labelled *S. aureus* (green) and coagulase-negative staphylococci (red). Cell nuclei of tissue stained with DAPI (blue). Scale bar 15 µm. **A** Optilene mesh from an open inguinal hernia repair excised for chronic pain and mesh shrinkage. Image shows biofilm on a mesh strand (blue autofluorescence of the mesh) containing *S. aureus* and coagulase-negative staphylococci within the biofilm. PCR results were concordant for both bacteria. **B** Ultrapro mesh from an open inguinal hernia repair excised for chronic pain. Image shows a biofilm containing abundant *S. aureus* colonies and fewer coagulase-negative staphylococci. PCR results were concordant for both bacteria*.* **C** TiMesh plug from an open inguinal hernia repair excised for chronic pain. Image shows biofilm on a mesh strand (blue autofluorescence of the mesh) containing predominantly coagulase-negative staphylococci*.* PCR results were positive for *S. epidermidis*. **D** Atrium mesh (pale green autofluorescence) from a laparoscopic TEP repair excised for recurrence. Image shows biofilm containing both *S. aureus* and coagulase-negative staphylococci. PCR results were concordant for both bacteria

Fifteen (75.0%) mesh samples demonstrated confocal microscopic evidence of staphylococci biofilm attached to the surface of the mesh (Fig. 1). Three (15.0%) meshes were positive for *S. aureus*, 3 (15.0%) meshes were positive for coagulase-negative staphylococci and 9 (45.0%) meshes were positive for both (Fig. 1).

### PCR identification

Bacterial biofilm identification by PCR was positive in 17 (85.0%) mesh samples. Four (20.0%) mesh samples were positive for *S. aureus*, 4 (20.0%) were positive for *S. epidermidis* and 9 (45.0%) were positive for both. When the two techniques were combined there was concordance with both confocal and PCR-bacterial biofilm identification positive for one or both bacterial species in 13 (65.0%) mesh samples (Fig. 1). Bacterial biofilms were identified by either confocal or PCR identification in 6 (30.0%) mesh samples and only 1 (5.0%) mesh sample was negative from bacterial biofilm by both techniques.

## Discussion

In this retrospective observational study, we investigated the presence of bacterial biofilms in 20 hernia meshes that have been explanted due to one or more clinical complications (i.e. pain, mesh shrinkage, hernia recurrence). No mesh was explanted for infective symptoms. Common skin bacterial species, i.e. *S. aureus* and coagulase-negative staphylococci/*S. epidermidis*, were targeted in our investigation as these bacteria are commonly implicated in biofilm infections of surgical implants [3, 11, 13]. Staphylococci biofilms containing one or both of these bacteria were identified in 95% of the specimens examined by either microscopic and/or DNA diagnostic techniques (Fig. 1). There was concordance with bacterial biofilm being identified with both microscopic and molecular techniques in 13 (65%) samples. Only one sample did not demonstrate evidence of staphylococci biofilm presence with neither microscopic nor DNA diagnostic techniques.

These results suggest that bacterial biofilms are readily found on inguinal hernia repair surgical mesh explanted due to clinical complications of shrinkage, hernia recurrence and pain. This is the first demonstration of biofilm on mesh from inguinal hernia repair and supports the findings of Kathju et al. of biofilm on mesh from ventral hernia repair [18]. Whilst our findings do not demonstrate a causal relationship, they do suggest that bacterial biofilm infections need to be considered in the pathophysiology of mesh-related complications. Bacterial biofilms can have various effects on the outcome of hernia repair surgery and appear to have the ability to reduce the effective pore size of mesh [1]. This likely leads to mechanical instability of the mesh, specifically under pressure-related impacts like coughing, which stresses the need for adapted biomechanical requirements and testing of meshes [19, 20]. Consistent with other surgical implants, such as orthopaedic prostheses [13], breast implants [14, 15] and many other surgically inserted devices, bacterial biofilm infections have been demonstrated to be an important causative mechanism in the non-septic failure in most, if not all, surgical implants investigated to date [3, 5, 6, 10, 13]. However, in the area of hernia mesh failure, this mechanism has not been investigated despite a plethora of publications relating to biofilms in other medical devices. These findings will be important as biofilm infection and surgical implants, in particular orthopaedic and breast implants have resulted in improvements in implant technology, implant handling and surgical technique [14, 15, 21]. Surgical technique recommendations for these implants are designed to minimise the risk of implant contamination at time of surgical insertion and include the use of alcohol-base skin preparation or antibiotic-impregnated skin barriers, prophylactic antibiotics, changing gloves and instruments between the dissection and implant phase, minimal or no-touch handling of implants [14, 15, 21]. Further research is required to investigate the causal relationship between mesh–biofilm infection and how it affects the integrity of surgically inserted mesh and its complications, as well as the development of improved surgical techniques to minimise the risk of mesh–bacterial contamination at the time of surgical repair. Since monitoring for biofilms is a relatively new approach, standardisation is necessary to obtain more scientific evidence for causative conclusions [22].

This study has several limitations. The specimens used for this study were formalin-fixed, as is common practice for diagnostic histological processes, but not ideal for bacterial biofilm analysis. Bacterial biofilms are not readily characterised in standard histopathological and microbiological techniques due to their unique three-dimensional EPS that makes identification and assessment of microbiological composition challenging. As such most techniques to identify bacterial biofilm (microscopic and DNA diagnostic techniques), are based on fresh tissue samples. Additionally, there was a significant time delay from time of explantation to investigation, resulting in less-than-optimal handling and storage of the mesh specimens for the diagnosis of bacterial biofilm infection. This may have resulted in degradation of the samples and affected the ability to extract DNA for identification and quantitation purposes. Importantly, the confocal imaging technique specifically identifies bacteria within biofilm, thus excluding the issue of contamination. Biofilm and the bacteria contained within it were clearly identified for an image positive result. This can be seen on the represented images from four specimens in Fig. 1. In all images the bacteria are fully contained within the three-dimensional EPS and in Fig. 1A and C the EPS is attached to mesh fibre strands that image as blue autofluorescence under the confocal conditions. Further investigation is required with ideal specimen handling and use of fresh tissue techniques to optimise the diagnosis of bacterial biofilm from clinical specimens to better understand the presence of biofilm on mesh at the time of mesh explantation. Similarly, the causative organisms require broader assessment to identify all bacteria implicated in mesh–biofilm infection and a causal relationship between biofilm–mesh infection and mesh complications needs to be determined to develop strategies for prevention and/or treatment.

Mesh has become an essential component of hernia repair; however, it is not as biologically inert as first considered, as it supports increased bacterial adherence as a function not only of the bacterial adhesion properties, but also on the textile, physicochemical properties and composition of the mesh [23]. The interaction between the biofilm pathogen and the host inflammatory response is complex and involves an alteration of the host environment [10]. Biofilm formation can be facilitated by the host inflammatory response because host inflammatory molecules facilitate adhesion to the surface of the device, particularly with *Staphylococcus* species [11, 24].

In 2012, Klinge and Klosterhalfen made a critical distinction between simple mesh porosity—the percentage area of mesh which is not covered by filaments, in contrast to the effective mesh porosity—representing only the area of “good” pores where bridging of scar tissue is avoided by sufficient inter-filamentary distance [25]. Furthering this concept, in 2019 Jacombs et al*.* determined that if the effective porosity is reduced due to mesh construct, surgical technique or axial loading such that it results in decreased mesh tissue integration, then contamination with biofilm formation may become significant and problematic [1]. In a vicious cycle, this increased biofilm formation in turn is likely to further reduce the effective porosity by blocking the remaining pores with the EPS coat.

Our findings, in conjunction to other similar studies, necessitate that general and hernia surgeons have a good understanding of bacterial biofilm disease including (1) how biofilm affects surgical implants; (2) the common bacteria involved in biofilm infections; (3) the role and mechanism of biofilm in implant failure and hernia mesh complications; (4) how biofilm infections can be prevented; (5) clinical presentations of biofilm-mesh infections; (6) diagnostic workup; (7) development of effective treatment (currently there is no effective treatment other than implant removal). An improved understanding of this relationship and the relevant underlying pathological mechanisms will underpin the development of biofilm-resistant mesh devices, prophylactic treatments and other important technological advancements to reduce hernia mesh complication rates in patients.

## Conclusion

The results of this preliminary study suggest that staphylococci biofilms may be linked to surgical implant complications of hernia mesh, such as unexplained pain, mesh shrinkage or hernia recurrence. Our findings highlight the pervasiveness of biofilm involvement in chronic mesh complications and failure. There is need for more studies on the topic to further characterise the underlying nature of this clinically significant relationship.
